# NOVEL FINDINGS TWO DECADES FOLLOWING COGNITIVE TRAINING: FINDINGS FROM THE ACTIVE TRIAL

**DOI:** 10.1093/geroni/igz038.1614

**Published:** 2019-11-08

**Authors:** Alden L Gross, George W Rebok, Walter Boot

**Affiliations:** 1 Johns Hopkins Bloomberg School of Public Health, Baltimore, Maryland, United States; 2 Florida State University, Tallahassee, Florida, United States

## Abstract

Although the only demonstrated panacea against cognitive decline, behavioral cognitive training usually fails to demonstrate transfer either to untrained cognitive abilities or to distal outcomes like everyday functioning. No such trials, however, have leveraged more than a decade of follow-up to adapt life-course perspectives. The Advanced Cognitive Training for Independent and Vital Elderly (ACTIVE) study remains the largest NIA-funded clinical trial of cognitively normal older adults. Efforts to re-invigorate the cohort after 20 years with external data linkages have coalesced around renewed interests in how training-related cognitive improvements affect long-term dementia risk, health care utilization and costs, credit scores, and active years in later life. The first presentation by Rebok overviews ACTIVE and its 20-year follow-up plans. Next, Gross and colleagues tested whether cognitive training attenuates the relationship between IADL difficulty and mortality; negative findings suggest proposed relationships between cognition and IADL difficulty are correlational, not causal, which has implications for transfer and trial outcomes. Third, Thomas tests a novel algorithm for classifying MCI, ushering opportunities to examine training effects on incidence of MCI after up to 20 years post-training. Marsiske then evaluates temporal transfer of training in reasoning ability, concluding that reasoning training and age, but not other baseline demographics, predict maintained alterations in reasoning abilities. Finally, Felix evaluates the stifling role of depressive symptoms on ability to benefit from training. Dr. Wally Boot, a recognized thought leader in transfer of cognitive training, will provide an illuminating discussion of promises and pitfalls of these lines of research.

